# Is There a Correlation Between Irritable Bowel Syndrome and Lactose Intolerance?

**DOI:** 10.7759/cureus.6710

**Published:** 2020-01-20

**Authors:** Ivan Cancarevic, Mahnoor Rehman, Beshoy Iskander, Sanee Lalani, Bilal Haider Malik

**Affiliations:** 1 Internal Medicine, California Institute of Behavioral Neurosciences and Psychology, Fairfield, USA

**Keywords:** irritable bowel syndrome, lactose intolerance, malabsorption, lactase deficiency, lactose-free diet, hydrogen breath test

## Abstract

Irritable bowel syndrome (IBS) is a poorly understood gastrointestinal disorder that affects a significant percentage of the population and has a strong negative effect on the quality of life. The lack of known pathophysiologic mechanisms has made finding effective treatment strategies difficult. One of the common recommendations by clinicians is a trial of a lactose-free diet. We have wondered if there was sufficient evidence in the currently available literature to support such a recommendation. We have also looked into other possible relationships between malabsorption syndromes and IBS. All the articles used for this review have been found in the PubMed database. We have taken into consideration the possibility that there may be both genetic differences and differences in the gut microbiome between populations living in different geographic regions. Therefore, we have included articles from different geographic regions to increase the generalizability of the findings. While there is a plethora of evidence that IBS patients commonly report milk intolerance, we have not found any conclusive evidence to suggest an objective link between IBS and any known malabsorption syndromes, including lactose malabsorption. Furthermore, trials of lactase supplementation have not led to clinical benefit. We concluded that there was no evidence to support routinely recommending a lactose-free diet for patients diagnosed with IBS, but including hydrogen breath testing in routine workup of IBS is a reasonable clinical decision. Ultimately, we believe that more clinical trials and chemical studies of the feces are needed to determine the pathophysiology and explore possible dietary recommendations for patients with IBS.

## Introduction and background

Functional digestive disorders, of which irritable bowel syndrome (IBS) is the most well known and best researched, are a common group of gastrointestinal (GI) disorders. The prevalence of IBS in the western populations is as high as 20% and accounts for 25% of visits to a gastroenterologist and up to 12% of visits to a primary care doctor [1-2]. A strong female predominance has been observed. The lack of known pathophysiologic mechanisms or specific symptoms makes diagnosing IBS a challenge. Patients are typically diagnosed with IBS when they fulfill the Rome IV criteria in the absence of alarm features (e.g., unintentional weight loss, anemia). The positive predictive value of such an approach is up to 98%. It has been associated with anxiety, somatization, fibromyalgia, and chronic fatigue. It is important to note that individual IBS-related complaints tend to respond to appropriate symptomatic management [3-6]. Talley and Spiller have found similar disturbances in the central processing of afferent signals to those seen in other chronic pain syndromes [7]. In a subset of patients, IBS symptom occurrence follows a bout of gastroenteritis, with more toxigenic organisms and longer illness duration increasing the risk. Some evidence points to a relationship between exaggerated serotonin release and IBS development in those patients [8].

Despite the apparent psychogenic nature of the disease, its shared characteristics with other conditions and its response to treatment, as described by Spiller and by Talley and Spiller, as well as correlation with infectious gastroenteritis, point to an at least partially organic etiology of the condition [5,7,8]. Furthermore, patients commonly report dietary triggers for IBS symptoms, including lactose and other polysaccharides [9]. Food intolerance is significantly more difficult to diagnose than food allergy due to the subjectivity of clinical symptoms. Moreover, it can be difficult to determine whether symptoms could be explained by a mechanism other than food intolerance (e.g., bowel distension after overeating or bloating due to overconsumption of gas-producing foods). Therefore, it is very difficult to give dietary advice to patients diagnosed with IBS [10]. In practice, many physicians recommend a trial of a lactose-free diet for all patients newly diagnosed with IBS.

Lactose is found in mammalian milk and is hydrolyzed by the enzyme lactase in the small intestine. The worldwide prevalence of hypolactasia is approximately 70% [11]. A study by Cloarec et al. showed that the quantity of lactose malabsorbed in France was about 60%; however, the majority of people studied showed no significant symptoms, and their daily consumption of milk and dairy products was not affected [12]. The symptoms of lactose intolerance are often difficult to distinguish from those of IBS, although patients who are lactose intolerant develop symptoms only after ingesting lactose-containing products. Taking into consideration the fact that the majority of people regularly consume lactose-containing products, it is often difficult to distinguish lactose intolerance from IBS. It is also worth noting that some foods and drinks may be incorrectly labeled. The severity of symptoms in lactase-deficient patients is dependent on the dose of lactose and lactase expression, as well as intestinal flora and sensitivity of the GI tract [13]. As is the case of patients with IBS, biopsies of patients with lactose intolerance tend to show no abnormalities, even in cases of clinically significant disease [14].

The purpose of this review is to explore the reported associations between lactose intolerance and IBS as well as the effect of dietary modification on IBS symptoms.

## Review

We will be analyzing a number of studies found in the PubMed database. Due to limited access to some of those articles, a full quality assessment could not be performed.

Current attitudes

In order to gain a deeper understanding of the role of dietary modification in IBS management, it is important to look at what has been clearly established so far and what clinicians currently recommend. It is also important to note that most patients receive empiric recommendations based on the experience of the provider rather than individualized recommendations based on specific guidelines. Dietary components can cause or exacerbate IBS symptoms in one-quarter of cases. Fiber supplementation has been frequently recommended despite limited clinical evidence to support its use. Insoluble fiber may, in some cases, even worsen symptoms [15]. Multiple studies have confirmed that a diet restricted in fermentable, poorly absorbed carbohydrates, sorbitol, and other sugar alcohols is beneficial [15-17].

Restriction of fermentable, poorly absorbed carbohydrates, sorbitol, and other sugar alcohols has led to clinical improvement in multiple studies, whereas fiber supplementation has not shown consistent benefit and has even caused symptom exacerbation in some patients. Considered together, it appears that it is not a lack of a necessary substance but rather an abundance of a harmful substance that leads to clinical symptoms. Patients may find such a restrictive diet difficult to adhere to; therefore, in this review, we are going to try to determine if the commonly recommended lactose-free diet could be comparably effective. Since lactose avoidance alone is a significantly easier dietary regimen to follow and lactose-free dairy products are widely available, if the lactose-free diet is found to be beneficial, it would be a reasonable alternative to more restrictive dietary regimens, especially in cases where adherence could be an issue. We are also going to look at the effect other carbohydrates may play in the development of IBS symptoms and whether restricting individual carbohydrates could be effective.

Self-reported milk intolerance

While some of the IBS symptoms can be evaluated objectively (e.g., diarrhea), many others are highly subjective. Furthermore, patients with IBS tend to have no abnormal findings on any of the traditionally used diagnostic tests (e.g., blood tests, colonoscopy, biopsy). Therefore, the patient’s own perception of the disease is often used to guide management. IBS patients commonly complain of lactose intolerance or, more frequently, milk intolerance. Multiple studies have been conducted to assess whether the perceived intolerance can be objectively measured. Saberi-Firoozi et al. surveyed 1,978 individuals above the age of 35 years in Shiraz, southern Iran, in order to determine the prevalence of symptoms of IBS and lactose intolerance. A total of 562 subjects reported lactose intolerance, with the prevalence being significantly higher in females, subjects taking NSAIDs or acetaminophen, and those reporting IBS symptoms. They recommended either a trial of lactose avoidance or a test for lactose malabsorption for patients suspected of having IBS [18]. Newcomer and McGill found lactase deficiency to be an uncommon cause of IBS among non-Jewish Caucasian patients of northern-western European background [19]. Several studies have attempted to correlate the reported milk intolerance with objective findings on hydrogen breath testing. Vernia et al. compared the prevalence of lactose malabsorption in patients diagnosed with IBS with that in patients with self-reported milk intolerance. The study involved 503 patients who fulfilled the Rome criteria for IBS and 336 subjects who reported milk intolerance. A hydrogen breath test was used to assess lactose absorption objectively. Patients with IBS were not found to be significantly more likely to have a positive test result (337 [66.9%] patients with IBS tested positive compared with 240 [71.4%] patients with reported milk intolerance). They concluded that there was a significant overlap between the two conditions, suggesting that hydrogen breath testing should be performed as part of the diagnostic workup for IBS and that lactose malabsorption may be clinically irrelevant in many patients with moderate milk consumption [20]. Vernia et al. also conducted a case-control study analyzing the hydrogen breath test results following a load of lactose in IBS patients with self-reported milk intolerance. Patients diagnosed with IBS without self-reported milk intolerance matched for age and sex were used as controls. The conclusion of the study was that self-reported milk intolerance does not help in identifying lactose malabsorbers. They claim that lactose is responsible for symptoms in a subset of IBS patients; however, they can only be identified by the occurrence of symptoms during the test [21]. Yang et al. compared lactose absorption in healthy volunteers, who were used as controls, and patients with a diarrhea-predominant form of IBS (D-IBS). They found that the risk of lactose intolerance was related to the dose of lactose ingested and intestinal gas production. It was increased in patients with D-IBS. Self-reported lactose intolerance was associated with avoidance of dairy products, whereas objective results of hydrogen breath testing were not. It should be noted that all the cases were diagnosed at the same hospital (Sir Run Run Shaw Hospital in Hangzhou, China) [22]. A study conducted by Gupta et al. in Northern India also found that while patients with IBS are more likely to report symptoms following lactose ingestion, the levels of breath hydrogen were similar to that in healthy subjects [23]. A different study by Rana et al. performed in the same population found that patients with the D-IBS have a higher incidence of lactose intolerance. However, the study involved only 25 patients and 25 controls [24]. Farup et al. found that IBS and lactose malabsorption were unrelated disorders in a Norwegian population and that milk-related symptoms and symptoms after lactose intake were unreliable predictors of lactose malabsorption. They suggested that precise symptom-based criteria might enhance the diagnostic accuracy of lactose malabsorption [25]. Varjú et al. performed a meta-analysis using a systematic literature search up to April 24, 2018, and also found that lactose intolerance, but not lactose malabsorption, was more frequent among patients with IBS compared with healthy controls. They believed that IBS was a possible contributing factor to lactose intolerance in people with lactose malabsorption [26].

A lot of clinically relevant information can be obtained from these studies. They clearly point to a high incidence of both lactose intolerance and IBS in the general population. The studies also clearly indicated that patients’ perception of lactose intolerance often does not correlate with the findings on the hydrogen breath test. There was also little evidence to suggest that objective lactase deficiency was more common among IBS patients compared with healthy controls. After lactose ingestion, IBS patients reported more symptoms, but breath testing did not yield a significantly higher percentage of positive results. Only one of the studies pointed towards a link between IBS and hydrogen breath test results. One of the major limitations of the studies analyzed is that most of the articles were geographically limited, raising questions about the generalizability of the findings. Taking into account the lack of clear pathophysiologic mechanisms behind IBS, the fact that different populations may have unique characteristics of the microbiome of their digestive tracts, and the possible presence of genetic differences between groups, findings that explain IBS symptoms in one population may not necessarily be universally applicable. Therefore, to reduce the possible bias, we selected articles from a number of different geographic regions. It is important to note that many of the clinical findings of IBS are highly subjective, making any studies analyzing IBS symptoms prone to bias. The prevalence of both IBS and lactase deficiency in the general population is high; therefore, it is reasonable to assume that the number of people suffering from both conditions simultaneously and independently is significant. The objective studies performed using the hydrogen breath test as the gold standard for diagnosing lactose malabsorption have found a discrepancy between the prevalence of symptoms of lactose intolerance and positive test results, suggesting that there may be other factors responsible for the occurrence of clinical symptoms in some patients. Lactase activity varies between individuals and so does the amount of lactose ingested. It is possible that in some cases, the slightly lower lactase activity combined with higher lactose load could overwhelm the enzyme activity, leading to clinical symptoms. The only study that found a difference in the prevalence of lactose malabsorption between IBS patients and healthy controls included only 25 cases and 25 controls, which could be explained by an error due to the sample size. It is also worth noting that patients usually report “milk intolerance” rather than lactose intolerance per se. A subset of patients may be intolerant to a substance other than lactose that is regularly found in milk. A possible way to resolve the issue could be to conduct a randomized clinical trial to compare the response to a lactose-free versus milk-free diet in patients with IBS and self-reported milk intolerance. It would also likely be beneficial to conduct more epidemiological studies, including an analysis of the differences in dairy consumption between different populations, as well as lactose content of those products.

Malabsorption of other carbohydrates and bacterial overgrowth

Considering that highly restrictive diets have most consistently led to clinical improvement, it is worth asking whether a carbohydrate other than lactose, such as fructose, may be responsible for clinical symptoms. Lack of any of the digestive enzymes could lead to malabsorption of an osmotically active or gas-producing substance, which could cause IBS symptoms. Bacterial overgrowth has also been proposed as a possible explanation of some of the IBS findings. It was also thought that intestinal bacteria (if the overgrowth is present) could interfere with the hydrogen breath testing, causing false-positive results in patients with IBS. Although testing every patient for possible malabsorption of such a vast number of substances as well as for bacterial overgrowth would not be realistic, finding any correlations could help explain at least some of the pathology of IBS. Rana and Malik claim that breath testing is an important step in IBS workup to evaluate not only for lactose malabsorption but also for carbohydrate malabsorption and bacterial overgrowth [27]. Corlew-Roath and Di Palma found that carbohydrate maldigestion has a similar incidence in patients with and without IBS; however, patients without IBS showed better response to dietary changes [28]. Wang et al. reported that small intestinal bacterial overgrowth had a limited impact on hydrogen breath testing and that the majority of patients with positive tests had lactose malabsorption [29]. A study by Zhu et al. suggested that hydrogen production and abdominal distension were not correlated with subjective bloating, likely indicating that the symptoms of IBS can be attributed to visceral hypersensitivity [30].

Taken together, these studies also clearly suggest that there is no significant association between malabsorption and IBS. The studies have also found no clear link between bacterial overgrowth and IBS, and no evidence was found to suggest that it was causing false-positive hydrogen breath test results. It is important to note, however, that of the patients with carbohydrate maldigestion, those without IBS showed a better response to treatment. Even symptoms such as bloating did not correlate with objective abdominal distension. The findings in these studies largely follow the trend seen in studies examining the role of lactose. Malabsorption incidence does not appear to be significantly higher in patients with IBS than in healthy controls. The most likely explanation of the clinical presentation is that patients with IBS who report food intolerance are more sensitive to certain dietary triggers rather than suffering from true malabsorption. It is also highly probable that IBS symptoms have multiple triggers, whereas patients intolerant to a single carbohydrate have only a single dietary trigger. That consideration could explain the difference in response to dietary changes, as reported by Corlew-Roath and Di Palma. The study by Zhu et al. likely answers many of the issues raised previously. Hypersensitivity to normal stimuli could explain the variability of clinical findings associated with IBS. It would also confirm that the origin of IBS is more similar to that of diseases such as fibromyalgia, compared with organic diseases of the GI tract. Finding the pathophysiologic mechanism behind the bloating sensation with no objective distension could help explain many of the neurological or psychological aspects of the disease.

Effect of dietary modification

In cases of genuine food intolerance, avoidance of dietary triggers leads to clinical improvement. As previously mentioned, dietary modification is usually empirically recommended to patients newly diagnosed with IBS. If IBS is at least partially caused by malabsorption or intolerance of certain foods, a clinical improvement upon dietary modification would be expected. Also, if an enzyme deficiency was responsible for the clinical presentation, enzyme supplementation should be beneficial. We have looked at a number of studies that tried to answer those questions. Lisker et al. performed a double-blind, cross-over study to compare the response of confirmed lactose malabsorbers to lactase and placebo. They found no association between symptom severity and lactase treatment, suggesting that IBS symptoms were independent of lactose maldigestion [31]. Another study attempted to compare the response of lactase-deficient patients with IBS to acidophilus milk with that of regular milk. The rationale was that acidophilus milk could “correct the imbalance of flora” as well as provide patients with bacterial lactase. The study showed that lactase-deficient patients were as intolerant to acidophilus milk as to unaltered milk [32]. Campbell et al. suggested that gut bacteria may be producing toxic metabolites as a result of anaerobic digestion of carbohydrates and other foods in the small intestine, possibly affecting signaling mechanisms and explaining food intolerances and IBS symptoms [33].

Overall, neither lactose supplementation nor acidophilus milk has led to clinical improvement. Campbell et al. concluded that there might be other toxic metabolites that are responsible for clinical findings in IBS. Those results largely follow the pattern seen previously. If IBS was caused by lactase deficiency presenting as lactose malabsorption, lactase supplementation would be expected to lead to clinical improvement, as is the case of patients with lactose intolerance as an independent clinical entity. Acidophilus milk supplied the gut with additional bacterial flora as well as bacterial lactase. The lack of clinical response in either of the studies suggests that objective lactase deficiency is not responsible for clinical findings associated with IBS, which follows the results of the studies conducted using hydrogen breath testing [31-32]. Campbell et al.'s suggestion that toxic metabolites may be associated with IBS symptoms and could provide an additional layer of understanding of the mechanisms of IBS beyond just visceral hypersensitivity. More studies need to be conducted to try to identify the substances produced in the gut of IBS patients, possibly including chemical studies of the feces.

## Conclusions

IBS remains one of the most common yet least understood clinical entities. Most clinicians prescribe treatment empirically. One of the frequently recommended approaches is a trial of a lactose-free diet. The purpose of this article was to assess whether the currently available literature supports such practice. We have also looked into some other dietary recommendations and their success in remedying IBS symptoms. Overall, the studies we have examined do not show any clear correlation between IBS and any defined malabsorption syndrome, including lactose intolerance. Moreover, lactase supplementation has not been an effective treatment. Visceral hypersensitivity or excessive production of harmful metabolites is more likely to explain the pathogenesis of IBS. Therefore, the literature we have obtained does not support routinely recommending a trial of lactose-free diet to all patients newly diagnosed with IBS. However, due to the similarity of clinical presentations of lactose intolerance and IBS and the high prevalence of both conditions in the general population, we believe that a hydrogen breath test should be performed in patients newly diagnosed with IBS in order to identify those who would be most likely to benefit from a lactose-free diet. Patients who report milk intolerance with no objective evidence of lactose malabsorption could be started on a trial of a milk-free diet rather than a lactose-free diet. Finally, we believe that more research into the pathogenesis and management of IBS is needed. Comparing the chemical analysis of the feces of IBS patients with that of healthy controls who consume a similar diet could help determine if the production of toxic metabolites was at least partially responsible for IBS symptoms. A randomized controlled clinical trial comparing lactose-free to dairy-free diet in patients with self-reported milk intolerance could help answer whether there is a substance other than lactose that is found in milk, and that may be a common trigger of IBS complaints.
